# N-terminal domain of tyrosyl-DNA phosphodiesterase I regulates topoisomerase I-induced toxicity in cells

**DOI:** 10.1038/s41598-023-28564-6

**Published:** 2023-01-25

**Authors:** Evan J. Brettrager, Selma M. Cuya, Zachary E. Tibbs, Jun Zhang, Charles N. Falany, Stephen G. Aller, Robert C. A. M. van Waardenburg

**Affiliations:** 1 Department of Pharmacology and Toxicology, University of Alabama at Birmingham, 155 Volker Hall, 1720 2nd Ave S., Birmingham, AL 35294 USA; 2 Department of Chemistry, University of Alabama at Birmingham, Birmingham, AL 35294 USA; 3 Present Address: Department of Molecular and Cellular Biology, Kennesaw State University, Kennesaw, GA 30144 USA; 4 Present Address: Cardiothoracic Surgery - Ascension Medical Group, 10580 North Meridian St. Ste 105, Carmel, IN 46290 USA

**Keywords:** Biochemistry, Molecular biology

## Abstract

Tyrosyl-DNA phosphodiesterase I (Tdp1) hydrolyzes phosphodiester-linked adducts from both ends of DNA. This includes the topoisomerase I (TOP1)-DNA covalent reaction intermediate that is the target of the camptothecin class of chemotherapeutics. Tdp1 two-step catalysis is centered on the formation of a Tdp1-DNA covalent complex (Tdp1cc) using two catalytic histidines. Here, we examined the role of the understudied, structurally undefined, and poorly conserved N-terminal domain (NTD) of Tdp1 in context of full-length protein in its ability to remove TOP1cc in cells. Using toxic Tdp1 mutants, we observed that the NTD is critical for Tdp1’s ability to remove TOP1-DNA adducts in yeast. Full-length and N-terminal truncated Tdp1 mutants showed similar expression levels and cellular distribution yet an inversed TOP1-dependent toxicity. Single turnover catalysis was significantly different between full-length and truncated catalytic mutants but not wild-type enzyme, suggesting that Tdp1 mutants depend on the NTD for catalysis. These observations suggest that the NTD plays a critical role in the regulation of Tdp1 activity and interaction with protein-DNA adducts such as TOP1cc in cells. We propose that the NTD is a regulatory domain and coordinates stabilization of the DNA-adducted end within the catalytic pocket to access the phosphodiester linkage for hydrolysis.

## Introduction

DNA topology is constantly under duress by processes associated with transcription, and replication during the cell lifespan^1–3^. To maintain genomic topological integrity, cells express a variety of DNA topoisomerases (TOPs). These enzymes utilize specific mechanisms that vary considerably to reverse topological constraints. However, they all share the key aspect that they generate a transient break in the DNA within the topoisomerase-DNA covalent complex (TOPcc) to alter the DNA topological configuration followed by religation of the DNA strand^1–3^. Stabilization of these transient TOPcc by local nucleotide perturbations or chemotherapeutics (e.g., camptothecin (CPT) targets TOP1cc) can lead to detrimental DNA lesions and eventual cell death^1^.

The DNA repair enzyme Tyrosyl-DNA phosphodiesterase I (Tdp1) catalysis the hydrolysis of phosphodiester linked DNA-adducts. Tdp1 removes a broad variety of DNA-adducts with different complexities^4–11^. These adducts include peptide- and protein-DNA adducts such as the Schiff-base linked PARP1 peptide bond to DNA and the naturally short-lived reaction intermediates formed by enzymes, such as TOP1, TOP2, and Tdp1 itself^12,13^. Moreover, Tdp1 can also remove smaller adducts such as oxidatively damaged nucleotides (e.g., 3’-phospho-glycolates), DNA incorporated ribonucleotides, and non-canonical nucleosides such as gemcitabine and fluorouracil^6,14–18^.

Tdp1 utilizes two highly conserved histidines to catalyze the hydrolysis of the phosphodiester linked DNA-adduct using a ‘hand-off’ mechanism. The first histidine is nucleophilic (His^nuc^; yeast His^182^; human His^263^) and attacks the phosphodiester linkage, releasing the adduct by forming a covalent bond with the DNA-end within a transient Tdp1cc. The second histidine acts as a general acid/base (His^gab^; yeast His^432^; human His^493^), facilitating the release of Tdp1 from the DNA end by activating a water molecule^4,8,13,19–25^. In contrast to the TOPs, Tdp1 catalysis does not mediate religation of the DNA-ends. The DNA-ends left by Tdp1 need additional processing by Polynucleotide kinase before DNA Ligase can religate the DNA strand. Thus, Tdp1 removes a potentially lethal DNA-adduct via the formation of another potentially toxic Tdp1cc and leaves a nicked strand behind. It is remarkable that the potential toxicity of a Tdp1cc is currently only highlighted by one mutant that causes a human pathology. The Tdp1His^493^Arg mutant forms the molecular basis for the rare autosomal recessive neurodegenerative disease, spinocerebellar ataxia with axonal neuropathy (SCAN1)^26,27^. Characterization of the Tdp1His^gab^Arg mutant and additional His^nuc^ and His^gab^ substitutions reveals that these catalytic mutants exhibit a lower overall enzyme activity due to a slower release of Tdp1 from its DNA complex, the basis of Tdp1 mutant induced toxicity^8,20,21,27–29^. Like the SCAN1 mutant, these cytotoxic catalytic mutants are recessive, showing that Tdp1 can remove another Tdp1 protein from its DNA-adduct *in trans*^8,20–23,27,29^.

**Figure 1:**
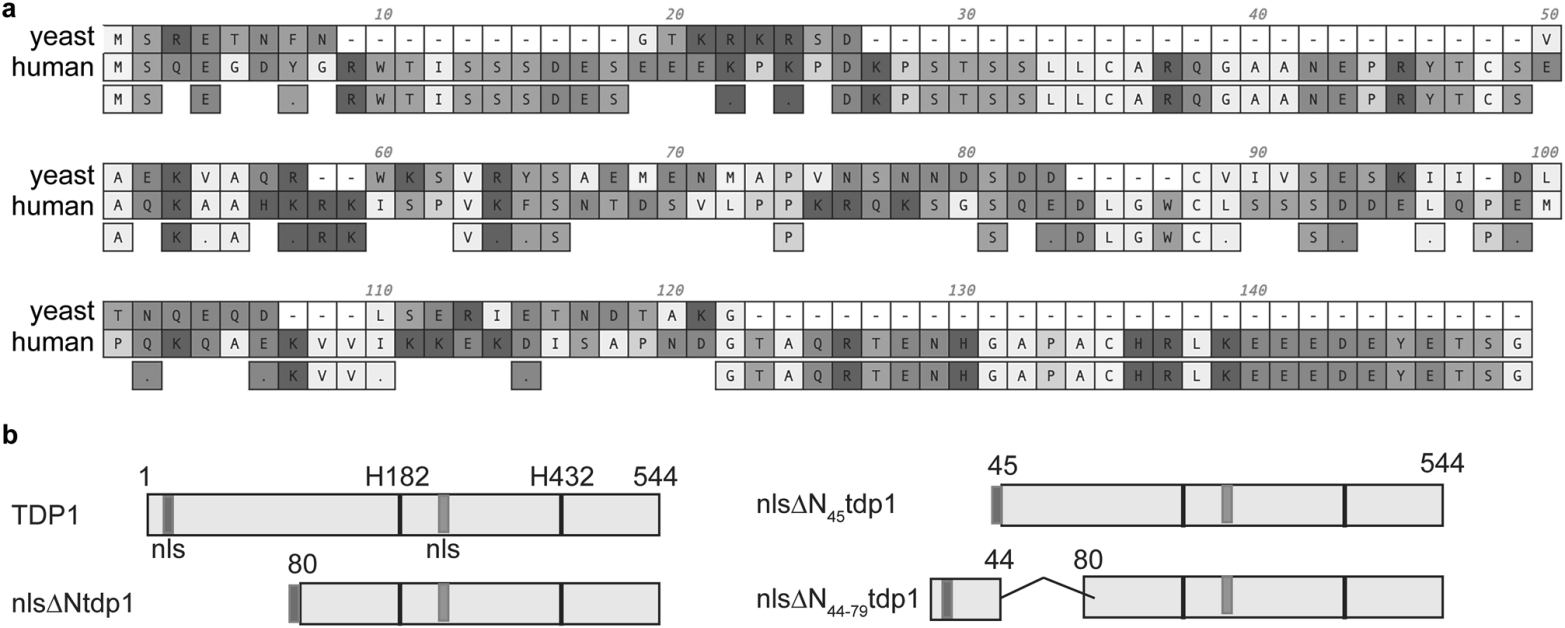
Tdp1 N-terminal domain sequence and constructs. (**a**) Comparison of yeast and human Tdp1 NTD amino acid sequence (alignment using MacVector 17.5.6). (**b**) schematic overview of N-terminal domain constructs used in this study. nls: nuclear localization sequence; ΔN: amino acid deleted region; numbers amino acid residue number; H: Histidine.

Over the last two decades, biochemical and structural studies provided a detailed understanding of the conserved chemistry of Tdp1 catalysis^4,8,19–21,24,30,31^. However, many of these studies are based on analyses of Tdp1 constructs that lack the structurally unresolved, poorly conserved, and functionally understudied N-terminal domain (NTD). Crystallography guided the definition of the Tdp1 NTD as amino acids 1–79 in yeast Tdp1 and 1–149 in human Tdp1 (Fig. 1a)^4,6–8,19–23,25,29,32–34^. The NTD was excluded in structural and biochemical studies due to proteolytic processing of the ectopically expressed full-length Tdp1 protein in bacterial and yeast cells^7,8,21,35^. Moreover, limited experiments comparing full-length, and N-terminal truncated human Tdp1 catalytic wild-type enzymes suggested that the NTD is dispensable for in vitro catalysis^20^. Additionally, these authors reported that the in vitro activity of truncated His^nuc^Ala and His^gab^Asn proteins is reduced at least 3000- to 15,000-fold compared to the wild-type enzyme, respectively. Conversely, the toxic phenotype we reported for expressing nuclear full-length catalytically active His^nuc^Ala and His^gab^Asn enzymes in cells suggests that these mutant enzymes are catalytically active, contradicting reports of biochemical in vitro activity of these respective N-terminal truncated mutant proteins^6,20,22,23^.

To study the function of the NTD in cells, we exploit previously characterized Tdp1 catalytic mutants that induce a toxic phenotype as readout for Tdp1 interaction with substrates and enzymatic activity in cells^8,21–23^. We utilize a *Saccharomyces cerevisiae* strain that is proficient in DNA damage response and repair, except for the *TOP1* and *TDP1* genes that are deleted (*top1Δ, tdp1Δ*)^8^. To prevent cellular adaptation towards the Tdp1-induced DNA damage, we use plasmid born *TDP1* constructs expressed from the galactose inducible *GAL1* promoter. We use elevated TOP1 protein levels to increase the number of TOP1cc as Tdp1 substrates, which results in similar phenotypes as yeast expressing endogenous levels of TOP1 treated with non-lethal concentrations of CPT^8,21,22^. Our observations suggest a potential mechanical role for the NTD in catalysis of TOP1cc in cells.

## Results

### N-terminal truncated Tdp1 mutants do not generate a TOP1-dependent toxicity

Yeast and human Tdp1s’ primary nuclear localization signals (nls) are positioned within their NTDs^36^. The yeast primary nls-sequence contains amino acid (AA) 8–17, while a functional secondary nls, AA 232–253, is located in catalytic domain (Fig. 1a). To examine the role of the Tdp1 NTD in hydrolysis of TOP1cc in cells, we generated N-terminal truncated constructs with the primary nuclear localization signal (nls) appended to the 5’-end (nlsΔN) of truncated constructs to ensure proper nuclear distribution (Fig. 1b)^8,21,22^.

**Figure 2:**
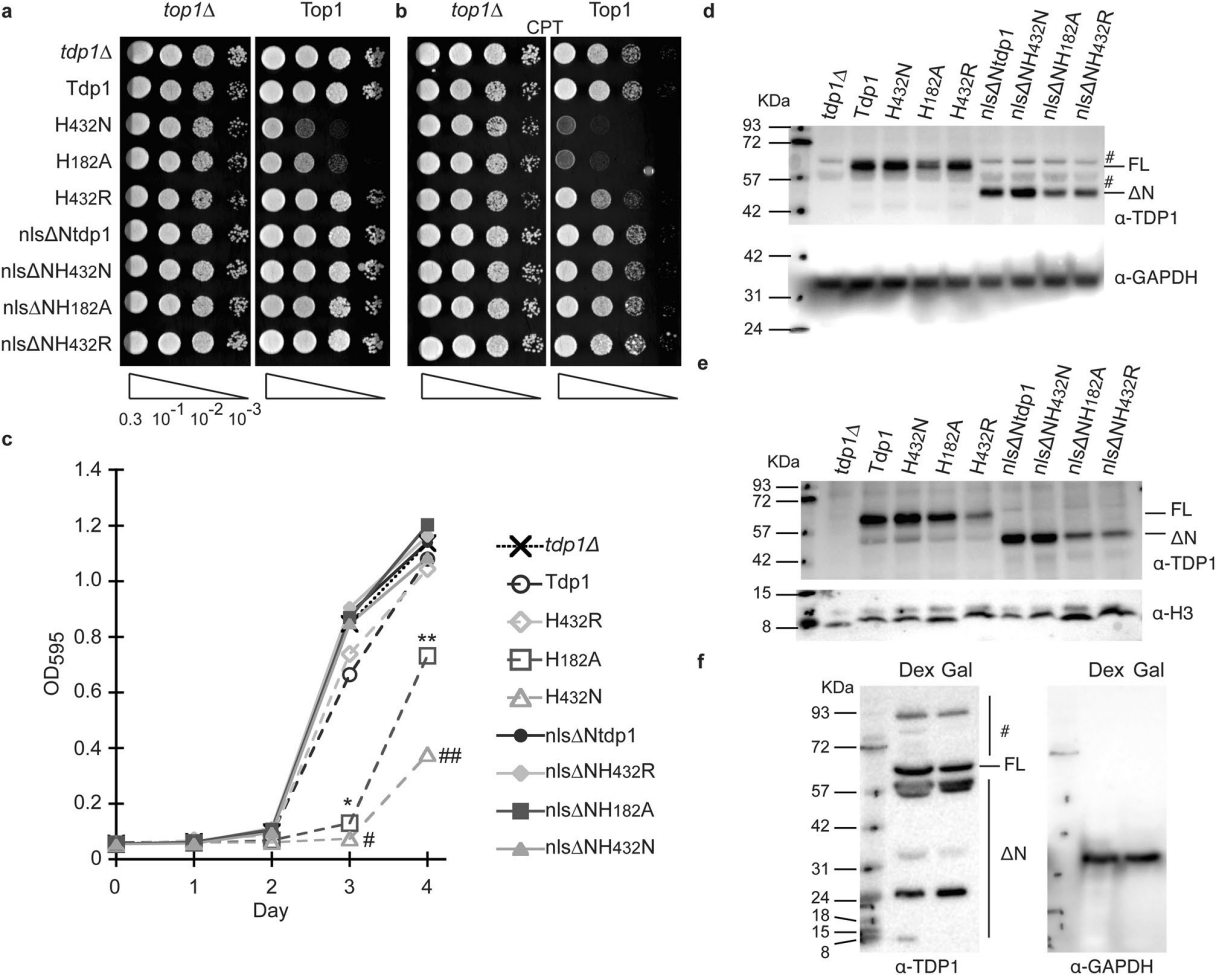
N-terminal domain is essential for in vivo Tdp1 catalysis of TOP1cc. (**a**) *top1*Δ, *tdp1*Δ cells were co-transformed with vector control (*top1*Δ) or YCpGAL1-*TOP1* U (Top1) and control vector (*tdp1*Δ) or the indicated YCpGAL1-*TDP1* L plasmid. Exponentially growing cultures were diluted to an OD_600_ of 0.3 and three times ten-fold serially (10^–1^, 10^–2^, 10^–3^, represented by triangle) diluted and spotted onto selective galactose plates, (a) without or (**b**) 5 with μg/ml camptothecin (CPT). Plates were incubated at 30 °C for 4 days. (**c**) Yeast transformants from (a-with Top1) grown in selective media supplemented with 2% raffinose were diluted to OD_600_ of 0.01 in selective media supplemented with 2% galactose and grown for 4 days at 30 °C. Every 24 h OD_600_ were determined from each culture. Average values of 4 independent growths are shown, SD is not shown for clarity with only Day 3 and Day 4 showing significant difference in 2-tail unpaired student T-test mutant versus *tdp1*Δ (vector control) for *tdp1*Δ-H182A (*D3: *p* = 2.5 10^–7^; **D4: *p* = 2.2 10^–5^) and *tdp1*Δ-H432N (#D3: *p* = 1.9 10^–8^; ##D4: *p* = 6.3 10^–4^). (d, e) Ectopically expressed TDP1 protein levels in 20 μg/lane total cell extracts (**d**) or nuclear extracts (**e**) from galactose induced transformants used in (a-vector control) resolved on 10% SDS-PAGE and stained with anti-TDP1, stripped and stain with anti-GPDH (d) or anti-Histone H3 (**e**). (**f**) Detection of endogenous TDP1 protein in 100 μg/lane of wild-type yeast total cell extracts cultured in dextrose or galactose supplemented media were resolved on 10% SDS-PAGE and stained with anti-TDP1, stripped and stain with anti-GPDH. FL: full-length TDP1; ΔN: N-terminal truncated TDP1 proteins. #: non-specific anti-Tdp1 antibody reactions; Molecular weight marker ladder in KDa. Shown are a representative agar plates (white box added for clear separation of the vector control (*tdp1*Δ) and Top1 expressing (Top1) spots) and immunoblots, cropped after adjustment of intensity of the whole plate/membrane using photoshop. All images shown are from one membrane or agar plate and are not merged products of different plate or membranes. Full membranes of immunoblots can be found in Fig. S1.

Colony formation assays of single transformants that express the full-length Tdp1 catalytic mutants displayed a TOP1-dependent toxicity range from high to low toxicity; H^432^N > H^182^A > H^432^R > Tdp1 (Fig. 2a)^4,8,23^. Conversely, expression of the analogous N-terminal truncated mutant proteins displays a similar viability as the truncated wild-type Tdp1 (Fig. 2a). Additionally, the truncated Tdp1 proteins did not affect viability after treatment with 1 μg/ml CPT in combination with elevated TOP1 protein levels that resulted in an enhanced toxicity in combination with the full-length Tdp1 mutants (Fig. 2b). The inability to induce a TOP1-dependent cytotoxic phenotype of the N-terminal truncated proteins was corroborated in a liquid culture viability assay (Fig. 2c).

We determined Tdp1 expression levels in total cell lysates and nuclear extracts. Analysis of galactose-induced total cell lysates and nuclear extracts did not show biological relevant differences between protein levels of full-length and N-terminal truncated Tdp1-alleles (Fig. 2d, e). While we detect some differences, for example, in nuclear extracts between the toxic full-length H^432^N and H^182^A, we also observe these differences between their truncated counterparts that do not induced toxicity (Fig. 2e). These observations suggest that the lack of TOP1-dependent toxicity between full-length and truncated Tdp1 proteins is not a result of a significant difference in expression levels.

Next, we explored whether the observed proteolytic cleavage of ectopically expressed Tdp1 proteins was an artifact of overexpression or was a physiologically relevant aspect of cells regulating detrimental effects of excessive Tdp1 activity. Western blotting of total cell lysates of the wild-type strain (*TOP1, TDP1*) cultured in dextrose and galactose media revealed a proteolytic cleavage pattern of endogenously expressed Tdp1 (Fig. 2f). Moreover, our N-terminal truncated proteins were similar in size to the endogenously generated truncated Tdp1 protein. This implies that ectopic and endogenous Tdp1 undergo a similar proteolysis process in yeast. Overall, these observations suggest that the Tdp1 NTD plays an important role in Tdp1 hydrolysis of TOP1cc in yeast cells with minor effects on Tdp1 protein stability.

### The complete N-terminal domain is essential for Tdp1 cellular function

**Figure 3:**
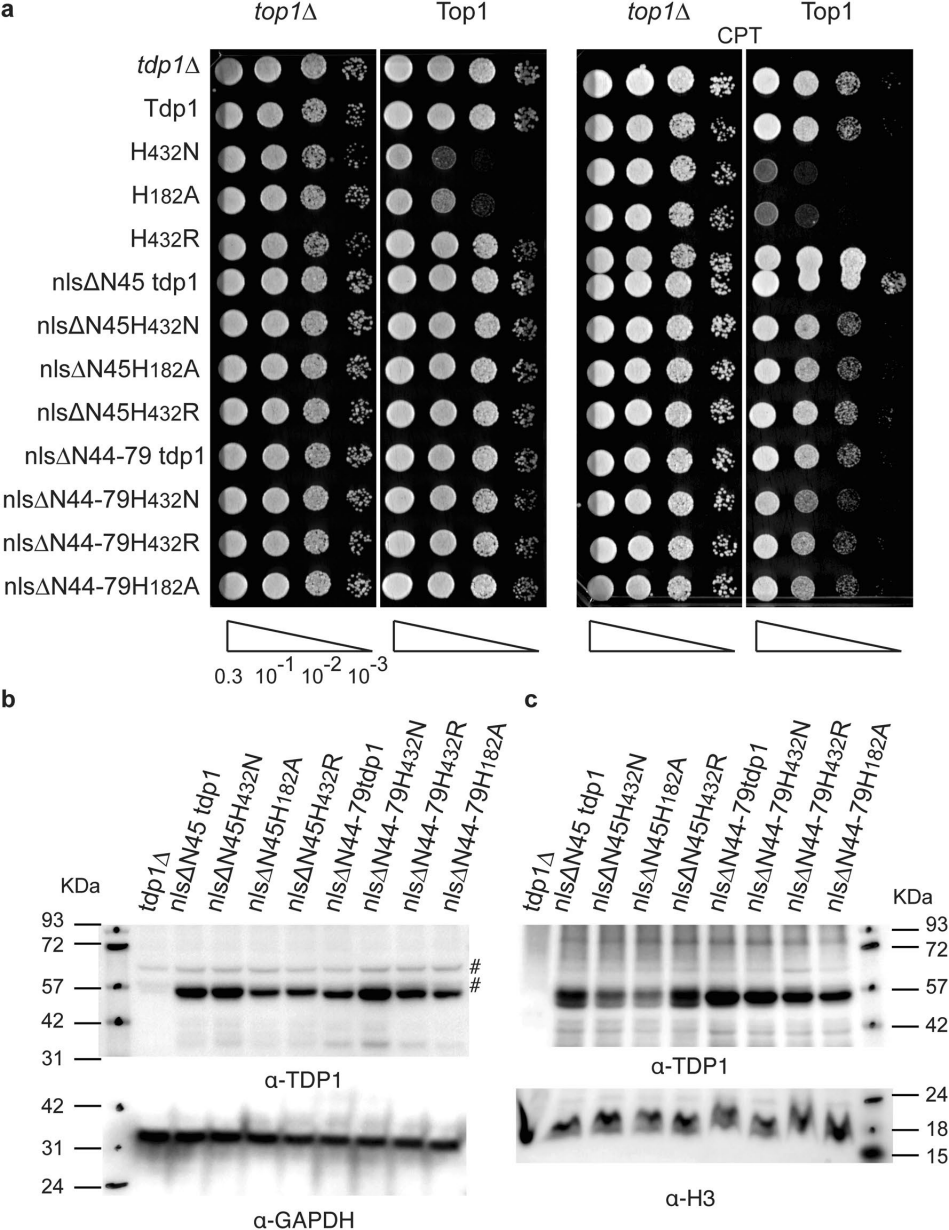
TDP1 in vivo catalysis of TOP1cc requires the complete NTD. (**a**) *top1*Δ, *tdp1*Δ cells were co-transformed with vector control (*top1*Δ) or YCpGAL1-*TOP1* U (Top1) and control vector (*tdp1*Δ) or the indicated YCpGAL1-*TDP1* L plasmid. Exponentially growing cultures were diluted to an OD_600_ of 0.3 and three times ten-fold serially diluted (10^–1^, 10^–2^, 10^–3^, represented by triangle) and spotted onto selective galactose plates, with or without 5 μg/ml camptothecin (CPT). Plates were incubated at 30 °C for 4 days. (**b**, **c**) Ectopically expressed TDP1 protein levels in 20 μg/lane total cell extracts (b) or nuclear extracts (**c**) from galactose induced transformants used in (a-vector control) resolved on 10% SDS-PAGE and stained with anti-TDP1, stripped and stain with anti-GPDH (b) or anti-Histone H3 (**c**). FL: full-length TDP1; ΔN: N-terminal truncated TDP1 proteins. #: non-specific anti-Tdp1 antibody reactions; Molecular weight marker ladder in KDa. Shown are representative agar plates and immunoblots, cropped after adjustment of intensity of the whole membrane using photoshop. All images shown are from one membrane or agar plates (white box added for clear separation of the vector control (*tdp1*Δ) and Top1 expressing (Top1) spots) and are not merged products of different plates or membranes. Full membranes of immunoblots can be found in Fig. S2.

To further investigate if the observed phenotypes were due to lack of specific sequence elements within the NTD, we generated two partial N-terminal truncations and appended the primary nls sequence to the 5’-end to ensure nuclear localization. We removed the first 45 amino acids of yeast Tdp1 NTD (nlsΔN45) and deleted amino acids 45–79 (nlsΔN44-79) (Fig. 1b). Expression of the nlsΔN45 and nlsΔN44-79 truncation mutants showed a similar viability as the nlsΔN-proteins and did not induce a toxic phenotype in a colony formation assay (Fig. 3a). Of note, the yeast nls is located within the first 45 amino acids and, therefore, we did not append the nlsΔN44-79 constructs with this nls sequence. As with the full N-terminal truncated (nlsΔN) protein, both partly truncated constructs were expressed (Fig. 3b) and located in the nucleus (Fig. 3c). However, the level of the nlsΔN44-79 proteins seem to be consistently slightly elevated in the nucleus compared to the nlsΔN45 proteins, possibly related to the presence of the native nls context. Overall, these results suggest that the complete N-terminal domain of Tdp1 is essential for Tdp1 mediated catalysis of TOP1cc in yeast.

### N-terminal truncated catalytic mutants show no toxicity in a *rad9* delete strain

**Figure 4:**
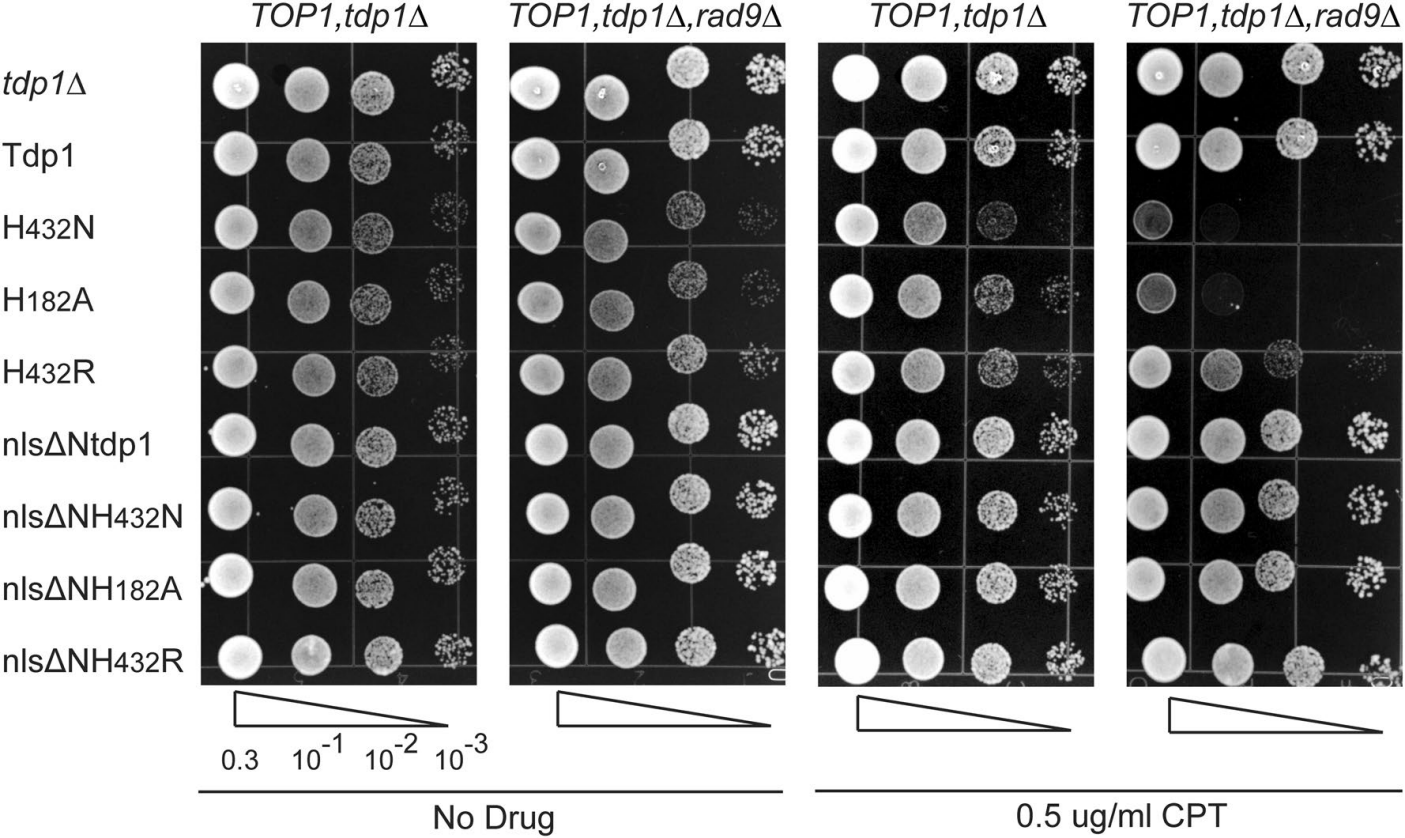
N-terminal truncated Tdp1 catalytic mutants do not reduce viability in the DNA damage impaired hypersensitive *rad9*Δ-strain. *TOP1*, *tdp1*Δ and *TOP1*, *tdp1*Δ, *rad9*Δ cells were transformed with control vector (*tdp1*Δ) or the indicated YCpGAL1-*TDP1* L plasmid. Exponentially growing cultures were diluted to an OD_600_ of 0.3 and three times ten-fold serially diluted (10^–1^, 10^–2^, 10^–3^) and spotted onto selective galactose plates, with or without 0.5 μg/ml camptothecin (CPT). Plates were incubated at 30 °C for 4 days. Shown are representative agar plates, cropped for clarity after adjustment of intensity of the whole plate using photoshop. All shown with and without CPT images are from one agar plate, respectively.

Unlike their full-length counterparts, expression of the N-terminal truncated Tdp1-mutants in yeast did not affect cell viability and CPT sensitivity. This lack of toxicity could be the result of reduced accumulation of nlsΔNTdp1cc due to decreased complex-stability. As such, we ascertained if cells impaired for the Rad9 DNA damage checkpoint protein could distinguish the potential cytotoxic lesions generated by these truncated Tdp1 mutants. Rad9 is a DNA damage response protein that signals for DNA damage checkpoint activation, resulting in activation of numerous DNA repair enzymes and arrest of cell cycle progression^37,38^. We previously reported that *rad9∆, tdp1∆, TOP1* cells display hypersensitivity to full-length Tdp1 H^432^N and H^182^A mutant enzymes but not the yeast H^432^R -SCAN1- mutant enzyme^22^. We concluded that the topology and/or stability of the Tdp1cc formed by the H^432^N and H^182^A mutants and the yeast H^432^R trigger different DNA damage responses^22^. Expression of the full-length catalytic mutants corroborated that loss of Rad9 exacerbates the TOP1-dependent toxicity with or without non-toxic (0.5 μg/ml) CPT levels for the H^432^N and the H^182^A mutants but not the H^432^R mutant (Fig. 4)^22^. However, the N-terminal truncated mutants did not induce a detectable TOP1-dependent toxicity with or without CPT. These observations suggest that the NTD of Tdp1 is essential to process TOP1cc in yeast cells.

### The N-terminal domain regulates mutant enzyme activity in vitro

**Figure 5:**
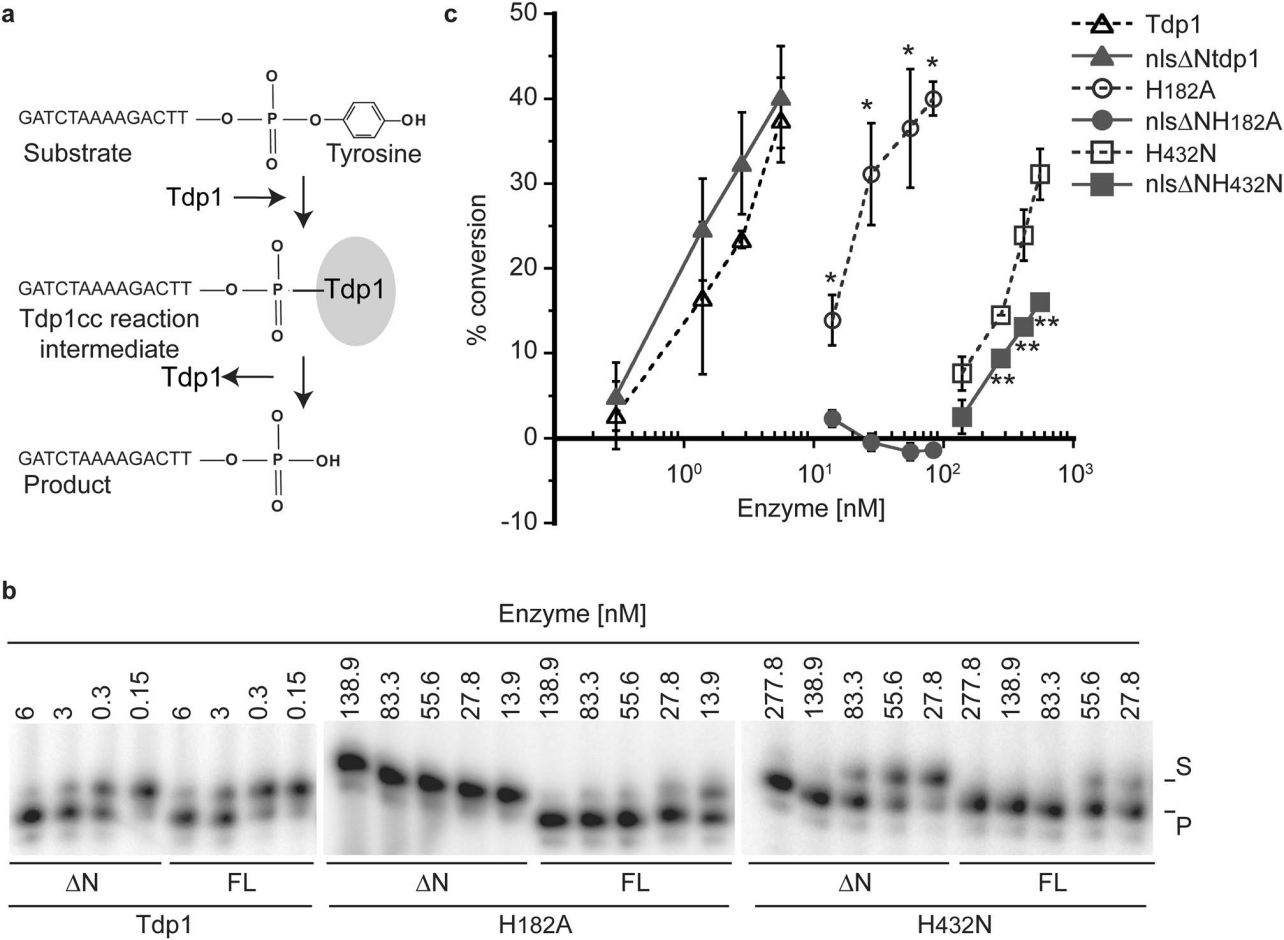
N-terminal domain affects TDP1 in vitro catalysis. (**a**) in vitro TDP1 catalytic activity assay reaction scheme. 14-mer oligonucleotide containing a 5’-^32^P label and a 3’phospho-tyrosine functions as substrate (S) which is covalently bound by TDP1 (TDP1cc) as reaction intermediate and is subsequently released as 3’phosphoryl Product (P). (**b**) N-terminal FLAG-tagged yeast TDP1 protein ranging from 5.6 to 333.3 nM was incubated with 16.7 nM of 5′-^32^P labeled substrate for 10 min at 30 °C, stopped and heat denatured. Reactions were was resolved on a denaturing 20% polyacrylamide/8 M urea gel, to detect the conversion of the 3′phospho-tyrosine (S) to 3′phosphoryl (P). Shown is a representative gel of each Tdp1 protein tested with full-length (FL) and N-terminal truncated (ΔN) proteins of the same catalytic site next to each other. Intensities were adjusted for the entire gel before cropping using photoshop. (**c**) The average and standard deviation of substrate to product conversion (product/ [product + substrate]) from (b) were quantitated by ImageStudio Lite (version 3.1.4, LI-COR) of at least three independent experiments. Standard deviations can be overshadowed by marker. 2-tailed unpaired student T-test full-length versus truncated: H^182^A* 0.0047 > *p* < 1.5 10^–6^; H^432^N ** 0.0015 > *p* < 0.0006. Shown are representative reaction resolved on 13 cm sequence gels, cropped for clarity after adjustment of intensity of the whole gel using photoshop. All images show full-length and truncated protein reactions resolved on same sequence gel next to each other and are not merged products of different sequence gels. Full sequence gels for each of the proteins in (**b**) can be found in Fig. S3.

There is a discrepancy between reported catalytic activity of N-terminal truncated Tdp1 mutant proteins and the phenotype when expressed in cells. For example, the human N-terminal truncated Tdp1H^nuc(263)^A mutant analog of yeast H^nuc(182)^A was originally reported to be catalytically inactive^20^. Nevertheless, we observed that the full-length yeast and human His^nuc^Ala enzymes are catalytically active and induce toxic phenotypes when expressed in yeast and human cells^8,22,23^. This suggests that the NTD plays an important role in facilitating Tdp1 catalysis. To further investigate potential differences between full-length and N-terminal truncated Tdp1 proteins, we purified to near homogeneity N-terminal FLAG-tagged full-length and N-terminal truncated proteins from yeast cultures for use in an in vitro single turnover activity assay^22,23^. Affinity purified full-length and N-terminal truncated FLAG-tagged wild-type, H^182^A and H^432^N Tdp1 proteins were incubated in reaction mixtures with a ^32^P 5’-end labeled 14-mer single stranded oligonucleotide with a 3’phospho-tyrosyl modification as the DNA-substrate. This 3’phospho-tyrosyl linkage represents the bond between the active site Tyrosine from TOP1 and the 3’phosphoryl site of the DNA that covalently binds TOP1 to the 3’-end of the DNA strand within a TOP1cc. Tdp1 activity is defined as removal of the tyrosine moiety of the 3’phospho-tyrosyl substrate over time, resulting in an often-undetectable transient Tdp1cc reaction intermediate and a final 3’phosphoryl product (Fig. 5a). After 10-min incubation at 30 °C, reactions were terminated, resolved by denaturing 8 M urea/20% polyacrylamide gel electrophoresis (PAGE) and quantified by PhosphorImage analysis of the polyacrylamide gels. The full-length and N-terminal truncated catalytic wild-type enzymes displayed a similar activity (Fig. 5b). Densitometry analysis showed that the truncated enzyme exhibits a consistent but nonsignificant, minor increase in activity over the full-length enzyme (Fig. 5c). Conversely, we observed a consistent significantly reduced activity for the H^432^N truncated versus full-length enzyme (Fig. 5b and c). A significant difference in single turnover activity was observed for the H^182^A mutant proteins: The N-terminal truncated H^182^A mutant protein is catalytically inactive (the detected + 2.3 to − 1.6 ‘relative activity’ is likely due to minor degradation of the oligonucleotide substrate that migrate about one nucleotide faster than the substrate band. This smaller substrate band appears to be processed by Tdp1 as a band migrating about one nucleotide faster than the anticipated phosphoryl-product is also detected), while the full-length enzyme exhibits catalytic activity (Fig. 5b and c). With the exception of the H^182^A mutant, our observations show that the N-terminal truncated enzymes are able to hydrolyze a 3’phospho-tyrosyl bond, albeit at a different rate than their full-length counter parts. As such the catalytically active truncated enzymes should have the potential to induce cytotoxicity when expressed in cells.

### Models of the yeast Tdp1 NTD imply a structured but dynamic entity adopting various conformations

**Figure 6:**
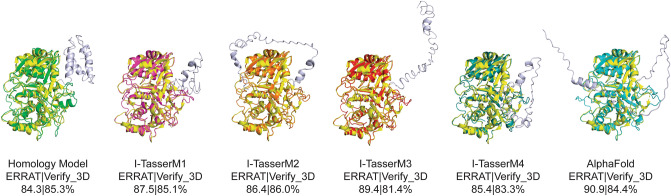
Predicted structural conformations of yeast Tdp1 N-terminal domain. The structure of the yeast Tdp1 NTD was predicted using homology modeling (Phyre2-server [http://www.sbg.bio.ic.ac.uk/~phyre2/html/page.cgi?id=index] followed by molecular modeling using MOE and AMBER99), AlphaFold (https://alphafold.ebi.ac.uk/) and I-Tasser (https://zhanggroup.org/I-TASSER/). Shown prediction models are overlayed on the yellow-colored yeast wild-type crystal structure of the catalytic core domain (missing NTD amino acid 1–79) PDB-1Q32^8^. All shown prediction models passed quality control of UCLA SAVESv6.0 server based on ERRAT-overall quality factor (statistics of non-bonded interactions) and a verify_3D score (compatibility of atomic model with its own aa sequence). All structure cartoons were generated using PyMol 2.5.2 (Molecular Graphics System, Schrödinger, LLC).

The structure of the NTD of Tdp1 has been unresolved due to proteolytic cleavage of this domain from ectopically expressed full-length Tdp1 in all cell models. Our observations demonstrate that the Tdp1 NTD plays a critical role in the processing of TOP1cc in yeast. To explore the structure of this domain we generated prediction models of the NTD of yeast Tdp1 using three different modeling platforms. As query to generate the prediction model we submitted the amino acid sequence to the Phyre2-protein fold recognition server (http://www.sbg.bio.ic.ac.uk/~phyre2/html/page.cgi?id=index)^39^. Residues missing between folded fragments were placed by molecular modeling using Molecular Operating Environment (MOE) [Chemical Computing Group Inc. 2013] and AMBER99^40^ energy optimization. The resulting model was subjected to molecular dynamic simulation (10 ns) followed by quality control by the UCLA SAVESv6.0 server (https://saves.mbi.ucla.edu/). The quality assurance of the predicted yeast Tdp1 model scored an ERRAT^41^-overall quality factor (statistics of non-bonded interactions) of 84.3 and a verify_3D score^42,43^ (compatibility of atomic model with its own aa sequence) of 85.3% (Fig. 6), suggesting a good quality prediction model. In addition, we also employed AlphaFold (https://alphafold.ebi.ac.uk/) and I-Tasser (https://zhanggroup.org/I-TASSER/) to generate prediction models of the yeast NTD. These two prediction platforms provided additional models of full-length yeast Tdp1 with more unfolded regions than the homology model generated with Phyre2/MOE-AMBER99 (Fig. 6). Not all I-Tasser models generated passed the SAVESv6.0 quality test. Figure 6 shows all models that passed the SAVESv6.0 ERRAT and verify_3D quality assurance scores. Overall, these prediction models show that the NTD of yeast Tdp1 is a globular-fold domain of a highly dynamic nature, suggesting the NTD maintains numerous conformations.

## Discussion

Tdp1 catalyzes the hydrolysis of a phosphodiester bond that covalently links adducts to the 3’- and 5’-ends of nicked DNA. These adducts range from damage nucleotides (e.g., 3’phospho-glyocate) to proteins or peptides covalently linked to a DNA-end such as TOP1. TOP1 catalysis depends on a transient TOP1cc linking TOP1 via a 3’phospho-tyrosyl bond to the 3’-DNA end. This enzyme–DNA complex can be trapped by local DNA perturbations (e.g., single strand break) or chemotherapeutics like camptothecin with topotecan and irinotecan as clinically active analogs^1^.

Tdp1 uses two catalytic histidine residues that facilitate a two-step reaction cycle centered on the formation of a transient Tdp1cc reaction intermediate. The vulnerability of this catalytic cycle was revealed by the Tdp1 mutation of His^gab^ to Arg (H^493^R) in patients with the rare autosomal recessive neurodegenerative disease SCAN1^27^. This SCAN1 mutant exhibits a reduced catalysis rate caused by a delay in the dissociation-step resulting in stabilization of the enzyme–DNA reaction intermediate^21,27,29^. Additional substitutions of either conserved catalytic histidine also result in a toxic phenotype due to an increased stability of the Tdp1cc^8,21–23^. Even so, Tdp1-induced toxicity is always dependent on the presence of a DNA-adduct, which is predominantly TOP1cc in our yeast studies.

We utilized the decreased turnover rate and toxic character of these Tdp1 mutants to ascertain the biochemical and cellular function of Tdp1 N-terminal residues. Amongst Tdp1 proteins the NTD is poorly conserved in sequence and size^20^, structurally unresolved and functionally understudied. This is a consequence of reports that human Tdp1 in vitro activity is independent of its NTD^20,33^. Furthermore, all resolved crystal structures of yeast and human Tdp1 are of N-terminal truncated proteins as a result of cellular processing of ectopic expressed Tdp1 proteins^8,21,24,35^. However, we discovered that this is not specific for ectopic expressed Tdp1 as we detected a similar degradation pattern for endogenous Tdp1 in yeast. Analogous to the crystal structures, Tdp1 catalytic chemistry was primarily studied using N-terminal truncated Tdp1 enzymes^4,6–8,19–21,24,29,30,33–35^. Yet, no additional studies were reported on the effects of losing the NTD on Tdp1 function (wild-type or mutant) in cells.

Over the last 10 years, potential roles for the NTD were reported. The NTD of hTdp1 is post-translationally modified by, among others, phosphorylation and SUMOylation^44,45^ and facilitates Tdp1-protein interactions with proteins such as PARP1 and XRCC1^46,47^. Of note, most of these interacting partners are specific for mammalian cells and have no obvious homologs in yeast. Yet these functional observations suggest at least a cellular role for the Tdp1 NTD.

Herein, we evaluated the role of the N-terminal residues (AA 1–79) in context of the Tdp1 full-length enzyme, with a focus on resolving nuclear TOP1cc and analyzing hydrolysis of the related 3’phospho-tyrosyl linkage in vitro. We generated different N-terminal truncated constructs appended with Tdp1’s own primary nls (AA 8–17) to ensure proper nuclear localization. Yeast Tdp1 catalytic mutant proteins lacking the NTD, or part thereof, did not elicit the TOP1-dependent toxicity that was generated by the corresponding full-length mutant enzymes. This lack of toxicity of the N-terminal truncated Tdp1 proteins was not due to gross differences in protein levels or lack of nuclear localization. Subsequently, we examined whether this lack of toxicity was a result of loss of catalytic activity. The catalytic wild-type full-length and truncated (nls∆N) enzymes exhibit similar in vitro activities, consistent with reports of hTdp1^7,20^. Intriguingly, densitometry showed that the truncated wild-type enzymes exhibit a slightly faster turnover rate that was consistent, but not significant, compared to the full-length enzymes. However, the reverse effect was observed for the H^432^N mutant enzyme where the truncated enzyme displayed a significantly reduced single turnover activity versus the full-length mutant enzyme. The influence of the NTD on Tdp1 catalysis was most significant for the H^182^A mutant proteins. The full-length H^182^A enzyme hydrolyzed the 3’phospho-tyrosyl bond while the truncated H^182^A protein lacked catalytic activity. The lack of activity of the yeast truncated H^182^A mutant is consistent with the reported lack of activity of the analogous N-terminal truncated human H^263^A mutant^7,20^. We previously reported that the catalytic activity of the full-length H^nuc^A mutant is facilitated by a conserved His residue adjacent to the His^nuc^^22,23^. The flexibility of the His^181^ residue, along with the ability of Tdp1 to hydrolyze DNA-adducts that differ substantially in size and complexity, indicates that the catalytic pocket of Tdp1 is very adaptive. Yet, His-mediated catalysis is driven by the need to stabilize the position of the phosphodiester linkage in catalytic pocket that we propose is coordinated by the NTD and non-catalytic residues within the pocket. Recent small-angle X-ray scattering (SAXS) of the full-length human Tdp1 protein showed that the N-terminal residues form a structural entity and not a random flexible chain of residues^48^. We attempted to replicate these SAXS results using the yeast Tdp1 full-length enzyme but failed due to protein aggregation at protein concentrations at 2 and 3 mg/ml under various salt and pH conditions. As such, we presume that the yeast Tdp1 NTD, like the human NTD, forms a dynamic entity that could be structured under certain circumstances. To support this presumption of a globular-fold of the yeast Tdp1 NTD we generated prediction models using three independent in silico structure prediction platforms (Phyre2/MOE/AMBER99, I-Tasser and AlphaFold). These three platforms predicted six potential NTD configurations that passed the SAVESv6.0 ERRAT and Verify_3D quality control. They indicate that the yeast Tdp1 NTD-structure can amend to numerous globular-conformations.

Based on the observations described herein, we propose that the NTD interacts with the free DNA tail protruding of the conserved single strand DNA binding groove that accommodates three additional nucleotides upstream the adducted nucleotide located in the catalytic pocket^24^. This NTD-DNA tail interaction would alleviate the tail movement that facilitate the coordination of the position and stability of the adducted nucleotide in the catalytic pocket. This ‘NTD-DNA stabilization’ model is supported by the recently resolved crystal structure of two human N-terminal truncated (lacking first 149 AA) Tdp1 enzymes bound to the single strand ends of a ss-ds-ss hybrid oligonucleotide^34^. In this crystal structure, the two Tdp1 proteins coordinate the position and stability of the longer DNA substrate within the catalytic pocket of each other; in other words, one core-molecule acts as the ‘NTD’ of the other core-molecule.

This potential role of the NTD, to position and facilitate stability of the DNA-adduct within the catalytic pocket, emerges as essential for the H^182^A mutant. The H^182^A catalytic pocket experiences a dramatic change as His^181^ rotates into the catalytic pocket to function as alternative nucleophilic histidine^22^. This requires repositioning of the DNA-adduct within the catalytic pocket to enable hydrolysis of the phosphodiester linkage^22,23^. Without the NTD, the predicted uncontrolled movements of the DNA tail would prevent correct and stable positioning of the adducted DNA-end impairing catalytic activity.

Although this proposed mechanistic model might explain the catalytic activity of the N-terminal truncated Tdp1 proteins, it does not explain why the yeast expressing truncated mutant enzymes do not induce a TOP1-dependent toxicity that is generated by the full-length enzyme. Hence, it appears that in cells, the NTD in its entirety is not only necessary for Tdp1 mediated hydrolysis of TOP1cc but also critical for fulfilling additional roles. One potential additional role for the NTD could include Tdp1 recruitment (translocation) stimulated via post-translational modification and/or TDP1-protein interactions^36,44,46^. Moreover, our preliminary data with human N-terminal truncated mutant Tdp1 enzymes suggest that Tdp1 induced cytotoxicity in human cells is also dependent on the presence of the NTD (study in progress).

In sum, the data presented herein show that the NTD plays a critical role in the regulation of Tdp1 activity and interaction with TOP1ccs in cells. We propose that the NTD coordinates stabilization of the DNA-adduct within the catalytic pocket to access the phosphodiester bond for hydrolysis. In addition, removal of the NTD provides a potentially more direct mechanism to control Tdp1 cellular activity to process TOP1ccs as an alternative to the known regulation of Tdp1 enzyme levels via the transcription-translation rate and proteasomal protein degradation.

## Materials and methods

### Yeast strains, plasmids, and drugs

*Saccharomyces cerevisiae* strain used: KWY3 [*TOP1*, *tdp1∆*] (*MATα, ura3-52∆::LoxP, his3∆200, leu2∆1, trp1∆63, tdp1∆::sd*), KWY4 [*top1∆*, *tdp1∆*] (KWY3, *top1∆::HIS5*), ECY2 (KWY4, *pep4∆::TRP1*, *prb1∆::URA3*) and ECY4 [*TOP1, tdp1∆, rad9∆*] (*MATα, ura3-52, his3∆200, leu2∆1, trp1∆63, rad9::hisG, tdp1∆::URA3*)^21,22,49^. Tdp1 (YCpGAL1-Tdp1·L (*LEU2*) and TOP1 YCpGAL1-yTOP1·U (*URA3*) were expressed via the galactose-inducible promoter *GAL1* as described in Gajewski et al.^21^. *TDP1* mutant alleles were created using QuikChange Site-Directed Mutagenesis kit (Stratagene/Agilent). The different N-terminal truncated *TDP1* alleles were created using PCR. All generated *TDP1*-alleles were confirmed by DNA sequencing. pRS416 and pRS415 were used as control vectors. Dimethyl sulfoxide (DMSO) and CPT were obtained from Sigma (St. Louis, MO). All PCR primer sequences are available upon request. All experiments were independently repeated at least three times.

### Semi-quantitative colony formation

Semi-quantitative colony formation assays were performed as described in Gajewski et al.^21^. Briefly, single colony cultures of co-transformed yeast with indicated vectors were diluted to OD_600_ 0.3 in TE and tenfold serial diluted. 5 μl of each dilution was spotted onto selective media plates containing 2% galactose with indicated concentration of 1 μg/ml CPT, 0.025% DMSO and 25 mM HEPES (pH 7.2). Plates were analyzed after 4 days at 30 °C using a G:Box imager and the accompanied GeneSynV1.6.1.0 software (Syngene). For presentation, orientation and intensities (by correcting brightness and contrast) of whole images were modified in Adobe Photoshop before cropping and figures were finalized (adjusted in size) in Adobe Illustrator. At least three independent experiments of two single colony independent repeats were assayed.

### Liquid culture viability assay

Single colonies of yeast cells transformed with the indicated vectors were grown in selective media with 2% dextrose overnight at 30 °C, diluted 1/50 in 2% raffinose selective media for overnight growth at 30 °C. Cultures were diluted to OD_600_ of 0.001 in 2% galactose selective media and cultured for 4 consecutive days at 30 °C. OD_600_ was determined every 24 h. Four independent experiments of single colony were assayed and for significance analysis 2-tailed unpaired student T-test was used of a Tdp1 expression vector relative to vector control (*tdp1∆).*

### Immunoblotting of yeast cell extracts

Immunoblotting was performed as described in Gajewski et al.^21^. Briefly, we used anti-yTdp1^8^, anti-Histone H3 (Abcam), and anti-GAPDH (Gentex) antibodies. Transformed yeast cells with the indicated vectors were grown in selective media supplemented with 2% dextrose the first night, then 1/100 diluted into selective media supplemented with 2% raffinose. Raffinose cultures were induced for 6 h with 2% galactose, corrected to the same OD_600_ and lysed with acid-washed glass beads (Sigma) at 4 °C in 50 mM Tris pH 8.0, 2 mM EDTA, 2 mM EGTA, 1 mM PMSF, 10% glycerol and Complete EDTA-free Protease Inhibitor (Roche). Lysate protein concentrations were determined by Bradford-assay (Bio Rad). Lysates were boiled in SDS buffer for 10 min and samples were loaded onto a 10% Bis–Tris PAGE in SDS buffer, blotted onto PVDF and immunostained with anti-yTdp1, stripped (62.5 mM Tris, 2% SDS and 0.8% β-mercaptoethanol) and reprobed with anti-GAPDH or anti-Histone H3 antibodies. Immunostaining was visualized by chemiluminescence using a G:Box imager and the accompanied GeneSynV1.6.1.0 software (Syngene). For presentation, orientation, and intensities (by correcting brightness and contrast) of whole gel images were modified in Adobe Photoshop before cropping and figures were finalized (adjusted in size) in Adobe Illustrator.

### Cellular fractionation

Cultures of 4–8 yeast colonies transformed with the indicated vectors were grown in selective media with 2% dextrose overnight at 30 °C, diluted 1/50 in 2% raffinose selective media for overnight growth at 30 °C. Cultures were diluted to an OD_600_ of 0.45 for 6 h in 2% galactose selective media. Cells were harvested and then resuspened in 48 mL Zymolyase Buffer (50 mM Tris pH 7.5, 25 mM EDTA, 1 M sorbitol, 30 mM DTT). 500 μl of 20 mg/ml zymolyase was incubated with 500uL 100 mM PMSF per sample for 15 min at room temperature. 1 mL zymolyase/PMSF mix was added to cells and incubated at 37 °C to digest cell walls and create spheroplasts. Pellets were resuspended in Harvest Buffer (20 mM HEPES, pH 8.0, 10 mM NaCl, 1 mM EDTA, 1 mM EGTA, 1 mM DTT, 2PI) and lysed with a Dounce Homogenizer. Cells were centrifuged to pellet the nuclei. The supernatant fraction was transferred to a microcentrifuge tube and following centrifugation, to clear any debris, the supernatant (cytosolic fraction) was transferred to a new microcentrifuge tube. The nuclear pellet was resuspended in Harvest Buffer (2 0 mM HEPES, pH 8.0, 10 mM NaCl, 1 mM EDTA, 1 mM EGTA, 1 mM DTT, 2PI) and centrifuged to pellet the nuclei. The supernate was subsequently discarded, and the pellet resuspended in Buffer C (10 mM HEPES, pH 8.0, 550 mM KCl, 100 mM EDTA, 100 mM EGTA, 1 mM DTT, 0.1% NP-40, 2PI). The sample was then vortexed and sonicated to break apart the nuclei. The lysate was then centrifuged, and the supernate (nuclear extract) placed into a new microcentrifuge tube. At least three independent experiments were assayed.

### Tdp1 protein purification from yeast

N-terminal Flag-tagged yTdp1 and hTdp1 alleles were cloned into YEpGal4-10GAL1·L vector and transformed into ECY2. Transformed yeast cells were grown at 30 °C in selective media supplemented with 2% dextrose the first night, 1/100 diluted into selective media supplemented with 2% raffinose. Raffinose cultures were induced with 2% galactose for 6 h before harvesting. Cells were harvested and protein purified as described in Gajewski et al.^21^. Briefly, cell extracts in HEE/2PI (50 mM HEPES, pH 8.0, 5 mM EDTA, 5 mM EGTA) were loaded onto SP Sepharose fast flow matrix (GE Life Sciences) and eluted with HEE/2PI 0.4 M NaCl, then affinity purified over anti-Flag M2 affinity matrix (Sigma). Flag-Tdp1 was eluted with 1 mg 3X Flag peptide in TEEK/2PI (50 mM Tris, pH 7.5, 1 mM EDTA, 1 mM EGTA, 100 mM KCl, 1 mM DTT). Tdp1 collected in the flow-through and concentrated in an Ultracel-30 K concentrator (Millipore). Protein concentration was determined using Bradford-assay (Bio Rad). Purity was checked by 10% Bis–Tris SDS-PAGE followed by Sypro Ruby staining and immunoblotting.

### Tdp1 in vitro activity assay

Activity assays were performed as described in Gajewski et al.^21^. Briefly, 14-mer (5’-GATCTAAAAGACTT-3’) oligonucleotide with a 3’-phosphotyrosine were used as substrates and identical oligos with a 3’-phosphate were used as substrate controls (Midland Certified Reagent Company, Inc.). Oligo was 5’-^32^P end-labeled as described in Gajewski et al.^21^. 302 fmol of oligo was incubated in reaction buffer (50 mM Tris, pH 7.5, 1 mM EDTA, 1 mM EGTA, 100 mM KCl, 2 mM DTT) with Tdp1 at specified concentrations in 18 µl for 10 min at 30 °C. 5 µl samples of reactions were combined with either 10 µl USB stop buffer/ 8 M Urea for analysis on 20% PAA/Urea sequencing gel, 2 µl SDS-PAGE sample buffer for analysis by 12% SDS-PAGE or 4 ul Bromophenol Blue/50% Glycerol. Assay gels were exposed on Phosphor-Imager screens and scanned with Storm 865 scanner (GE Life Sciences). Quantification of substrate conversion was performed with ImageQuant TL 1D version 7.0 (GE Healthcare). Graphs were generated in Kaleidagraph 4. For visualization, orientation, and intensities (by correcting brightness and contrast) of whole gel images were modified in Adobe Photoshop before cropping and figures were finalized (adjusted in size) in Adobe Illustrator. At least three independent reactions of each protein were assays.

## Supplementary Information

Supplementary Information.

## Data Availability

All data generated or analyzed during this study are included in this published article [and its supplementary information files]. The prediction models were generated using the following open sources: AlphaFold (https://alphafold.ebi.ac.uk/), Phyre2-server (http://www.sbg.bio.ic.ac.uk/~phyre2/html/page.cgi?id=index), and I-Tasser (https://zhanggroup.org/I-TASSER/). The protein sequence used was obtained from the Saccharomyces Genome Database (https://www.yeastgenome.org/locus/S000000427/sequence).
