# Supplementary material for: N-terminal domain of tyrosyl-DNA phosphodiesterase I regulates topoisomerase I-induced toxicity in cells
Source: Sci Rep. 2023 Jan 25;13:1377. doi: 10.1038/s41598-023-28564-6 (PMC9876888; doi:10.1038/s41598-023-28564-6)
Supplement: Supplementary file 1 — Supplementary Information. [file 41598_2023_28564_MOESM1_ESM.pdf]

Supplemental Figures

N-terminal domain of tyrosyl-DNA phosphodiesterase I regulates topoisomerase I-induced toxicity in cells

Evan J. Brettrager[1], Selma M. Cuya[1,2], Zachary E. Tibbs[1,3], Jun Zhang[4], Charles N. Falany[1], Stephen G. Aller[1], and Robert C.A.M. van Waardenburg[1]*

[1]Department of Pharmacology and Toxicology, University of Alabama at Birmingham, Birmingham, AL 35294

[2]Current address: Department of Molecular and Cellular Biology, Kennesaw State University1, Kennesaw, GA 30144.

[3]Current address: Cardiothoracic Surgery - Ascension Medical Group, 10580 North Meridian St. Ste 105, Carmel, IN 46290.

[4]Department of Chemistry, University of Alabama at Birmingham, Birmingham, AL 35294

*Address for correspondence: Robert C.A.M. van Waardenburg, 155 Volker Hall, 1720 2nd Ave S., Birmingham AL 35294-0019, USA. Fax: 001 205 934 8240, E-mail; rvanwaar@uab.edu.

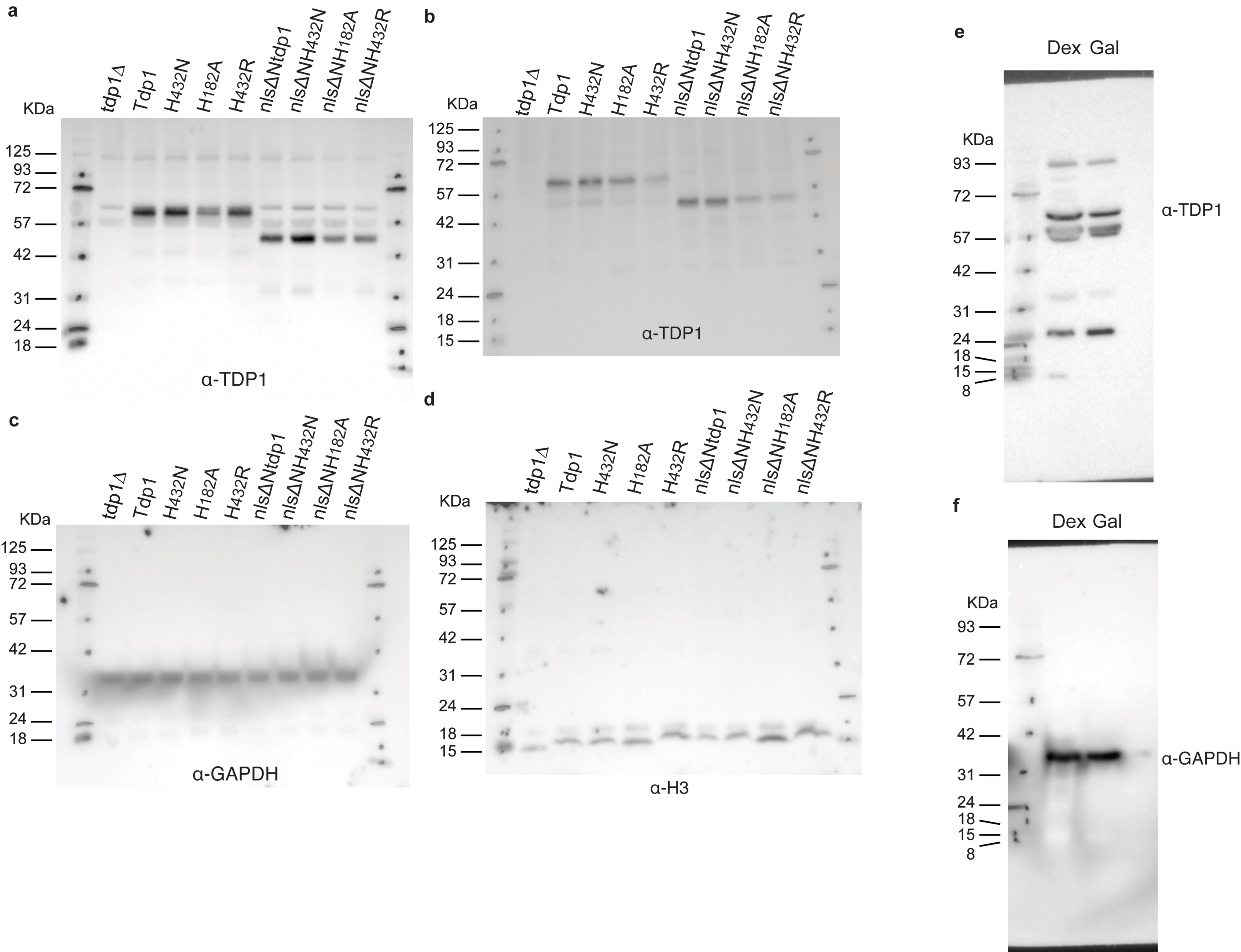
a
KDa
tdp1Δ
Tdp1
H432N
H182A
H432R
nlsΔNtdp1
nlsΔNH432N
nlsΔNH182A
nlsΔNH432R
125
93
72
57
42
31
24
18
α-TDP1
b
KDa
125
93
72
57
42
31
24
18
15
α-TDP1
c
KDa
α-GAPDH
d
KDa
α-H3
e
Dex Gal
KDa
8
α-TDP1
f
Dex Gal
KDa
α-GAPDH

Figure S1. Full western blots for N-terminal domain is essential for *in vivo* Tdp1 catalysis of TOP1cc (Fig. 2 in main text). *top1*Δ, *tdp1*Δ cells were co-transformed with vector control (*top1*Δ) and control vector (*tdp1*Δ) or the indicated YCpGAL1-*TDP1*•L plasmid. Ectopically expressed TDP1 protein levels in 20 μg/lane total cell extracts (a) and nuclear extracts (b) from galactose induced transformants resolved on 10% SDS-PAGE and stained with anti-TDP1 (a, b), stripped and stain with anti-GPDH (c) and anti-Histone H3 (d), respectively. (e, f) Detection of endogenous TDP1 protein in 100 μg/lane of wild-type yeast total cell extracts cultured in dextrose or galactose supplemented media were resolved on 10% SDS-PAGE and stained with anti-TDP1 (e), stripped, and stained with anti-GPDH (f). Molecular weight marker ladder in KDa. Shown are representative full immunoblots of the cropped immunoblots shown in Fig. 2d-f.

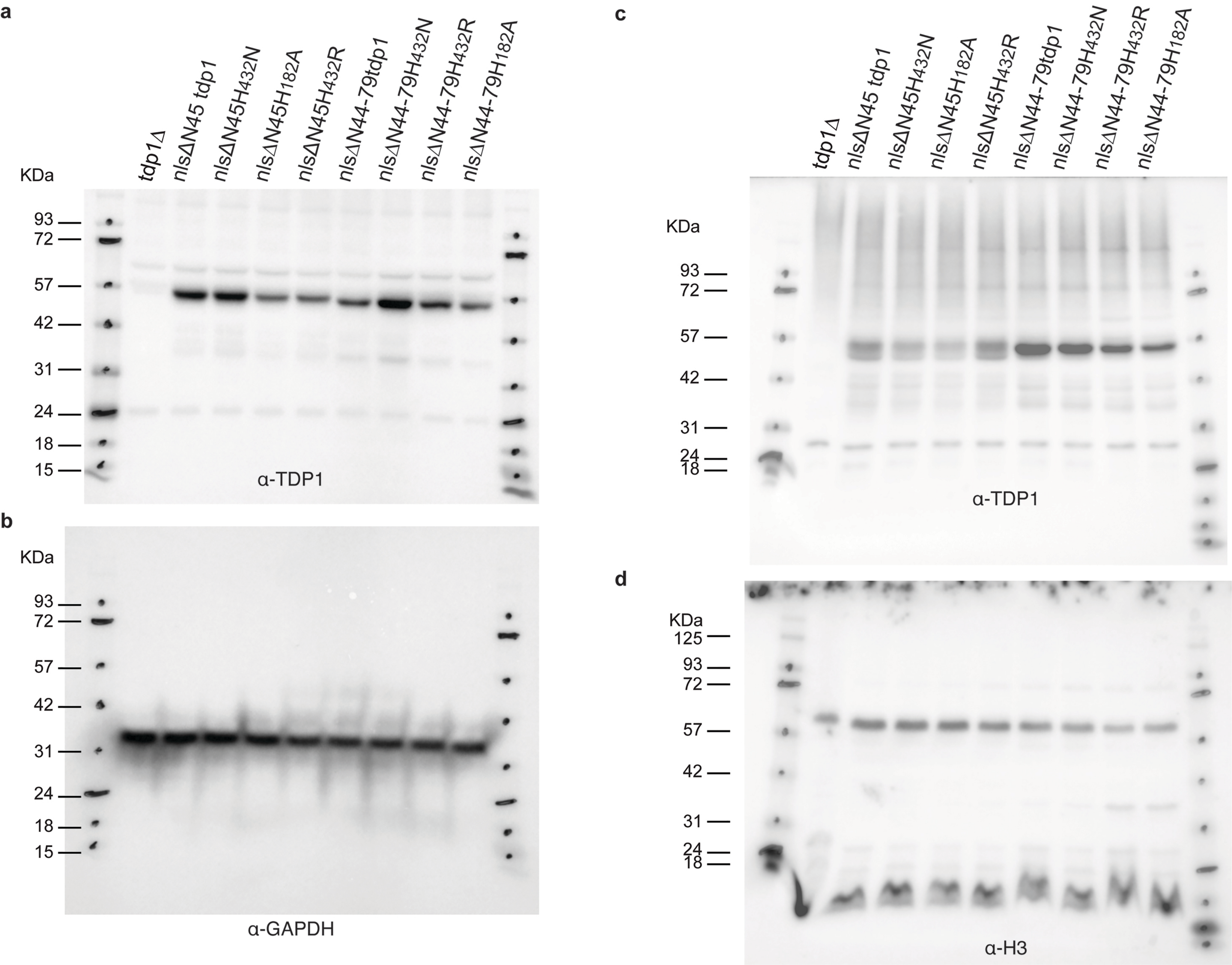
a
tdp1Δ
nlsΔN45 tdp1
nlsΔN45H432N
nlsΔN45H182A
nlsΔN45H432R
nlsΔN44-79tdp1
nlsΔN44-79H432N
nlsΔN44-79H432R
nlsΔN44-79H182A
KDa
93
72
57
42
31
24
18
15
α-TDP1
b
KDa
93
72
57
42
31
24
18
15
α-GAPDH
c
tdp1Δ
nlsΔN45 tdp1
nlsΔN45H432N
nlsΔN45H182A
nlsΔN45H432R
nlsΔN44-79tdp1
nlsΔN44-79H432N
nlsΔN44-79H432R
nlsΔN44-79H182A
KDa
93
72
57
42
31
24
18
α-TDP1
d
KDa
125
93
72
57
42
31
24
18
α-H3

Figure S2. Full western blots for TDP1 in vivo catalysis of TOP1cc requires the complete NTD (Fig. 3 in main text). *top1*Δ, *tdp1*Δ cells were co-transformed with vector control (*top1*Δ) and control vector (*tdp1*Δ) or the indicated YCpGAL1-*TDP1*•L plasmid. Ectopically expressed TDP1 protein levels in 20 μg/lane total cell extracts (a) and nuclear extracts (c) from galactose induced transformants resolved on 10% SDS-PAGE and stained with anti-TDP1 (a, c), stripped and stain with anti-GPDH (b) and anti-Histone H3 (d), respectively. Molecular weight marker ladder in KDa. Shown are representative full immunoblots of cropped immunoblots in Fig. 3b, c.

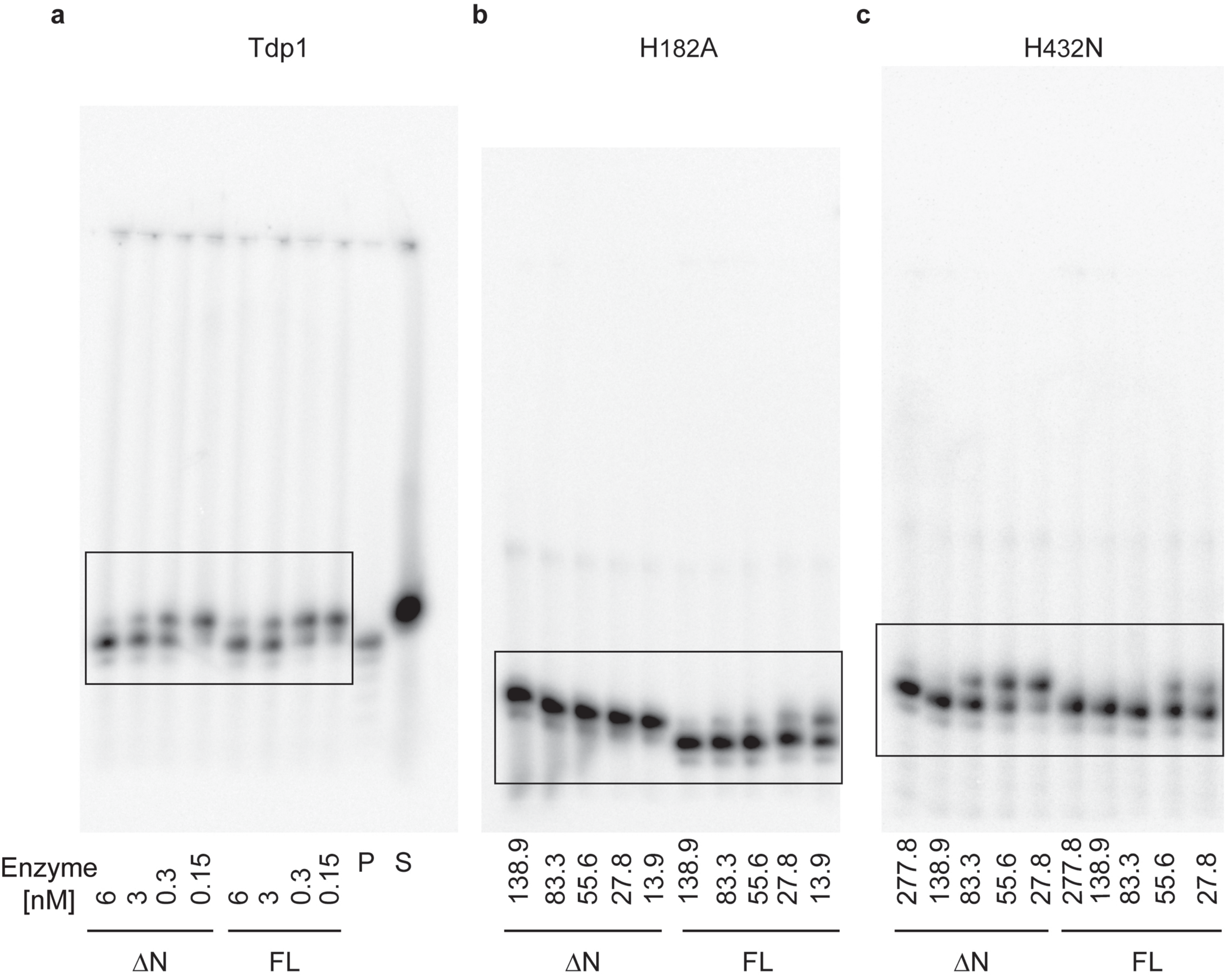

a
Tdp1
Enzyme [nM]
6
3
0.3
0.15
6
3
0.3
0.15
P
S
ΔN
FL
b
H182A
138.9
83.3
55.6
27.8
13.9
138.9
83.3
55.6
27.8
13.9
ΔN
FL
c
H432N
277.8
138.9
83.3
55.6
27.8
277.8
138.9
83.3
55.6
27.8
ΔN
FL

Figure S3. Full sequence gels for N-terminal domain affects TDP1 *in vitro* catalysis (Fig. 5 in main text). N-terminal FLAG-tagged yeast TDP1 protein ranging from 5.6 to 333.3 nM was incubated with 16.7 nM of 5′-^{32}P labeled substrate for 10 minutes at 30 °C, stopped and heat denatured. Reactions were was resolved on a denaturing 20% polyacrylamide/8M urea gel, to detect the conversion of the 3′phospho-tyrosine (S) to 3′phosphoryl (P). Shown is a representative uncropped sequence gel of each FLAG-Tdp1 protein. Activity assay with full-length (FL) and N-terminal truncated (∆N) proteins of the same catalytic site next to each other in the same gel. (a) Tdp1, (b) H182A, (c) H432N. Box represents the cropped figure shown in Fig. 5b.
